# Allogeneic Stem Cell Transplantation for Adult T-Cell Leukemia/Lymphoma—Romanian Experience

**DOI:** 10.3390/jcm9082417

**Published:** 2020-07-28

**Authors:** Alina D. Tanase, Andrei Colita, Oana G. Craciun, Lavinia Lipan, Zsofia Varady, Laura Stefan, Adela Ranete, Sergiu Pasca, Horia Bumbea, Mihaela Andreescu, Viola Popov, Alexandru Bardas, Daniel Coriu, Anca Roxana Lupu, Ciprian Tomuleasa, Anca Colita, Olivier Hermine

**Affiliations:** 1 Department of Stem Cell Transplantation, Fundeni Clinical Institute, 022328 Bucharest, Romania; alinadanielatanase@yahoo.com (A.D.T.); oanagabrielacraciun@yahoo.com (O.G.C.); lipan.lavinia@yahoo.com (L.L.); varadyzsofia@gmail.com (Z.V.); laura.stefan@icfundeni.ro (L.S.); adela_carciumaru@yahoo.com (A.R.); ancacolita@yahoo.com (A.C.); 2 Department of Hematology, Titu Maiorescu University of Medicine, 040441 Bucharest, Romania; 3 Department of Hematology, Colțea Hospital, 030171 Bucharest, Romania; anca.lupu@yahoo.com; 4 Department of Hematology, Carol Davila University of Medicine and Pharmacy, 020021 Bucharest, Romania; horiabum@gmail.com (H.B.); daniel.coriu@umfcd.ro (D.C.); 5 Medfuture Research Center for Advanced Medicine, Iuliu Hatieganu University of Medicine and Pharmacy, 400349 Cluj Napoca, Romania; pasca.sergiu123@gmail.com; 6 Department of Hematology, University Hospital, 050098 Bucharest, Romania; 7 Department of Hematology, Colentina Hospital, 020125 Bucharest, Romania; tevetmihaela@gmail.com (M.A.); violamariap@gmail.com (V.P.); 8 Department of Hematology, Fundeni Clinical Institute, 022328 Bucharest, Romania; bardas.alexandru@yahoo.com; 9 Department of Hematology, Ion Chiricuta Clinical Cancer Center, 400015 Cluj Napoca, Romania; ciprian.tomuleasa@umfcluj.ro; 10 Department of Hematology, Iuliu Hatieganu University of Medicine and Pharmacy, 400349 Cluj Napoca, Romania; 11 Department of Petriatics, Carol Davila University of Medicine and Pharmacy, 020021 Bucharest, Romania; 12 Service d’Hématologie Adultes, Hôpital Universitaire Necker-Enfants Malades, Assistance Publique Hôpitaux de Paris, Université Paris Descartes, 75015 Paris, France; ohermine@gmail.com; 13 Institut Imagine, INSERM U1163, 75015 Paris, France

**Keywords:** allogeneic stem cell transplantation, adult T-cell leukemia/lymphoma, Romanian experience, case-series, European cohort

## Abstract

Adult T-cell leukemia/lymphoma (ATLL) is a rare and aggressive mature T-cell malignancy caused by the human T lymphoma virus I (HTLV-I) affecting 3–5% of HTLV-1 carriers and is usually diagnosed in endemic regions. Romania is a region with high prevalence of HTLV-1 infection and ATLL and with low median age at diagnosis for aggressive types. We performed a retrospective analysis of post-transplant outcome in the first Romanian patients with ATLL receiving hematopoietic stem cell allotransplant. The study population included eight patients (three males, five females), with median age of 39.5 (range 26–57), with acute (one case) and lymphoma type (seven cases) that received peripheral stem cells (PBSC) from matched related (MRD) and unrelated donors (MUD) after reduced intensity conditioning. Graft versus host disease (GVHD) developed in six patients. Relapse occurred in four cases (50%) at a median time of 5-months post-transplant. Six patients died: four cases with disease-related deaths and two patients with GVHD-related deaths. The median survival post-transplant was 19.5 months (range 2.3–44.2 months). The post-transplant survival at 1-year was 62.5%, at 2-years 50%, and at 3-years 37.5%. In our opinion allogeneic transplant improves outcome in aggressive type ATLL.

## 1. Introduction

Adult T-cell leukemia/lymphoma (ATLL) is a rare and aggressive mature T-cell malignancy caused by the human T lymphoma virus I (HTLV-I). ATLL develops in approximately 3–5% of HTLV-1 carriers and is usually diagnosed in endemic regions that include the south-west region of Japan, Central and South America, central Africa, the Middle East, Far East, central Australia, and Romania [1,2,3]. ATLL diagnosis is based on clinical features, serum anti-HTLV-1 antibody and ATLL cell morphology, and immunophenotype [4,5]. The current disease classification (Shimoyama classification) defines four clinical subtypes based on the presence of organ involvement, leukemic manifestation, high lactate dehydrogenase (LDH) and hypercalcemia: acute, lymphoma, chronic (favorable and unfavorable), and smoldering [6]. The treatment strategy is based on the aforementioned classification. Thus smoldering and favorable chronic types are defined as indolent-type ATLL and are managed by active monitoring or zidovudine/interferon (AZT/IFN-α) with or without arsenic trioxide (ATO), while acute, lymphoma and unfavorable chronic types are defined as aggressive-type ATLL and may benefit from the association of chemotherapy, allogeneic hematopoietic stem cell transplant (allo-HSCT), and newer therapeutic agents [1,4,7]. The outcome of aggressive ATLL is poor in most patients not receiving allo-HSCT with 16–42% complete remission (CR) rate, followed by early relapses after a median progression-free survival time of 5- to 7-months and 4-year overall survival below 10% [1,5,8]. Several retrospective analyses of allo-HSCT in Japan and Europe reported long-term survival (at 3-years) in 27–45% of allo-HSCT recipients after chemotherapy but with significant associated morbidity and mortality [8,9,10,11,12]. As responses to intensive chemotherapy are not durable and the progressive disease has a poor outcome even after allo-HSCT, it is recommended that patients with aggressive ATLL should be referred as soon as possible to a transplantation center in order to receive up-front allo-HSCT, preferably from a HTLV-1 seronegative donor [1].

Romania is one of the regions with the highest prevalence of HTLV-1 infection in Europe (5.33 at 10,000 blood donors in 2003–2008) [3], therefore the prevalence of ATLL is also higher with lower median age at diagnosis (42.5 years) of aggressive types [13].

The aim of this study was to evaluate the post-transplant survival, relapse rate (RR) and treatment-related mortality (TRM) in a cohort of eight patients with ATLL that received allo-HSCT in a single transplant center.

## 2. Case-Series

The current study is a retrospective analysis of the ATLL cases that received allo-HSCT at the Fundeni Clinical Institute between January 2016 to July 2019. The current study was performed in accordance with the Declaration of Helsinki and with the approval of the Fundeni Clinical Institute ethics committee (24908/07.05.2020).

A total of eight patients (three males, five females) were included in the current case-series. The patients were diagnosed and initially treated in several centers across Romania before their referral to the transplant center. The median age at diagnosis was 39.5 years (range 26–57). According to Shimoyama classification, one case (12.5%) had acute type, while the rest had lymphomatous type. Five patients (62.5%) presented extranodal involvement, with three of those showing skin and bone marrow involvement as well, while one patient had multiple organ involvement (lung, kidney, parotid) and the fifth had meningeal involvement.

The primary end point of the study was post-transplant survival, defined as the time from the date of allo-HSCT until date of death from any cause or date of last follow-up. Causes of death were categorized into disease-related or treatment-related deaths. Disease-related deaths were defined as deaths caused by relapse or progression of ATLL among patients who survived for at least 30 days after transplantation. Treatment-related mortality (TRM) was defined as any death other than disease-associated deaths. Neutrophil recovery was considered for an absolute neutrophil count >0.5 × 10^9^/L for three consecutive days after transplantation. Acute and chronic graft versus host disease (GVHD) were assessed using traditional criteria [14,15]. Data were analyzed using summary descriptive statistics (medians and ranges) for continuous variables and numbers and percentages for discrete variables. Response criteria was assessed as described by Cheson et al. [16].

The clinical characteristics of patients are shown in Table 1.

All patients received initial chemotherapy as follows: six patients received CHOP regimen (cyclophosphamide, doxorubicin, vincristine, and prednisolone) and two cases received a modified version of LSG15 protocol (VCAP: vincristine, cyclophosphamide, doxorubicin, prednisolone; AMP-doxorubicin, ranimustine, prednisolone; and VECP: vindesine, etoposide, carboplatin, prednisolone) without ranimustine and with vincristine instead of vindesine [1,5,17]. In five cases antiretroviral therapy was added to chemotherapy. This was represented in three cases by interferon-α (IFN-α)/zidovudine (AZT) and in two cases by AZT. After first line treatment, three of the eight patients who were eligible for transplant had achieved complete remission (CR). The other five patients received second line therapy DHAP combination chemotherapy protocol (dexamethasone, high-dose cytarabine, cisplatin) two cases; modified LSG15 two cases; IFN one case) with achievement of CR in three case and stable disease (SD) in the other two. Median time from diagnosis to transplantation was 11.25 months (range 4.5–17.5 months). At time of transplantation six patients were in CR and two in SD. All patients received peripheral stem cells (PBSC) from matched related (MRD) as well as matched unrelated donors (MUD) after reduced intensity conditioning (RIC) with Fludarabine 30 mg/m^2^/day × 6 days-Busulfan iv 3.2 mg/kg/day × 2 days (Flu/Bu) or Thiotepa 5 mg/kg/day × 2 days-Fludarabine 40 mg/m^2^/day × 4 days-Busulfan iv 3.2 mg/kg/day × 2 days (TT/Flu/Bu), with four 4 cases in each as shown in Table 2. All donors had negative HTLV-1 serology. In one case (no. 5) a second haploidentical transplant was performed after early relapse following MUD transplant with progressive disease. Conditioning used for this procedure was Fludarabine 30 mg/m^2^/day × 4 days-Cyclophosphamide 14.5 mg/kg/day × 2 days-Total body irradiation 2Gy (Flu/Cy/TBI). Graft versus host disease (GVHD) prophylaxis consisted in cyclosporine A (3–5 mg/kg with threshold value 150–200 ng/mL) and methotrexate (15 mg/m^2^ on day +1, 10 mg/m^2^ on day +3, +6) +/− in all eight patients and post-transplant cyclophosphamide (PT-Cy; 50 mg/kg on day +3 and +4) associated with tacrolimus (0.015 mg/kg starting on day +5 to day +180) and mycophenolate mofetil (15 mg/kg from day +5 to day +100) following the procedure of haploidentical stem cell transplantation. Our GVHD prophylaxis protocol for ATLL with MUD transplants, did not include anti-thymocyte globulin (ATG). The GVHD prophylaxis was the same as sibling transplants. We assume that, in this extremely aggressive disease, the GVHD can be associated with a better survival. The median number of infused peripheral hematopoietic cells was 7.0678 × 10^6^ CD 34/kg (range 2.47–10.63 × 10^6^ CD 34/kg). All patients achieved engraftment with median time to neutrophil recovery of 17.87 days (range 15–21 days). Graft versus host disease (GVHD) developed in six patients (75%), three (50%) of which were acute GVHD and three (50%), chronic GVHD. All patients presented 100% donor chimerism at Day +30. Five patients were tested for HTLV-1 at 6-months post allogeneic HSCT and three of them (60%) presented a positive HTLV-1 viral load.

Relapse occurred in four cases (relapse rate of 50%) at a median time of 5-months post-transplant (range 2–25 months). In our cohort, six patients died during follow-up, the most frequent cause of death (four cases) was represented by relapsed/refractory disease (66.6%), one patient (16.6%) died because of metabolic imbalances and sepsis caused by chronic GVHD, and one (16.6%) because of refractory acute GVHD. The median survival post-transplant was 19.5 months (range 2.3–44.2 months). The post allo-HSCT survival at 1-year was 62.5%, at 2-years of 50%, and at 3-years of 37.5%.

## 3. Discussions

This retrospective study reports the outcome of first allo-HSCT performed for ATLL patients in our country. Romania is one of the regions with the highest prevalence of HTLV1 infection (5.33 at 10,000 donors in 2003–2008) [3]. Moreover, our group’s analysis on ATLL cases showed a lower median age at diagnosis in aggressive type ATLL (42.5 years) [13] compared to Japanese [6,11] as well as European studies [12]. This might reflect an origin of infection in the 1980s in Romania, where other outbreaks occurred by horizontal transmission in that period, including an outbreak of HIV-1 [18]. Our study group of patients receiving allo-HSCT was even younger with median age at diagnosis of 39.5 years (range 26–57). This emphasizes the need for efficient therapeutic approaches in a young population affected by a disease with poor outcome.

Patients with aggressive types of ATLL not receiving HSCT have CR rates of only 25–40%, median survival less than 1-year [17,19,20,21], and usually die of tumor progression. In our experience, in patients treated by chemotherapy between 2010–2019, CR rate was 16% and median overall survival (OS) was 6.5 months (5.1 for acute type, 8.0 for lymphomatous type) [13], compared to 3.5 months in acute type and 9.5 months in lymphomatous type in historical data reported by our group (oral presentation, data not published).

In the past two decades, several studies have shown improved OS in ATLL after allo-HSCT. Most publications are from Japan and present retrospective analyses of Japanese Registry Data. The largest retrospective analysis was performed on 578 patients with ATLL transplanted between February 1992 and December 2009 showing a 3-year OS of 36% with a median OS of 9–10 months and treatment-related mortality (TRM) of 34% [22]. The European Group for Blood and Marrow Transplantation’s Lymphoma Working Party (EBMT-LWP) published a retrospective EBMT Registry data analysis of ATLL cases with allo-HSCT and reported 3-year OS rate of 34.3% in a cohort of 17 patients [12]. There are no randomized studies comparing chemotherapy alone versus allo-HSCT consolidation published so far. Kawada et al. published a single center retrospective comparison of treatment outcomes in 29 patients with allo-HSCT versus 37 cases with chemotherapy showing a significative improved 3-year OS in transplant recipients (44.9 vs. 27.7%, *p* < 0.05) [23]. In our cohort the overall post allo-HSCT survival at 1-year was 62.5%, at 2-years 50%, and at 3-years 37.5%. The median post-transplant survival was 19.5 months (range 2.3–44.2 months).

Various studies have described a series of patient-related factors that could influence post-transplant outcome. Gender (male), age (>50 years), reduced performance status, and failure to achieve CR before allo-HSCT have been corelated with inferior outcomes [8,21,22,24]. Patients transplanted in first clinical remission and early, during the first 100 days after diagnosis (for related donors) are reported to have better outcome [21,25]. In a Japanese registry study on 386 patients, Hishizawa et al. reported a better survival probability for those transplanted in first CR (51%) compared to 26% in patients failing to achieve CR [8]. The report of The Revised ATLL International Consensus Meeting states that up-front allo-HSCT should be considered for suitable patients [1]. There are no data supporting the influence of ATLL subtype on post-transplant outcome [21,24].

Our patient population had a median age of 39.5 years (range 26–57). The only patient older than 50 years had a post allo-HSCT survival of 37.7 months. Two out of three male patients survived longer than 2-years. In our cohort, 6/8 patients were in CR prior to HSCT. Three of these patients achieved CR after first line chemotherapy and two of them survived for more than 44 and 37 months, respectively after HSCT. The other three patients were in CR after salvage chemotherapy, two of them having long term survival of 26.7 and 41 months, respectively. Both patients with SD at HSCT relapsed early after 2- and 7-months. Transplants were performed after a median time of 11.25 months (range 5–23.5 months) from diagnosis. It is remarkable that in our study all patient with post allo-HSCT survival longer than 2-years were transplanted after more than 11-months from diagnosis. All of them were in CR, emphasizing the importance of disease control before performing allo-HSCT.

Initial studies from Japan reported allo-HSCT performed mainly from MRD using bone marrow cells [26,27]. Sibling donors are available only for 1/3 of patients and a large proportion are HTLV-1 carriers, condition associated with higher disease-related mortality and donor derived ATLL, leading to the recommendation to preferably use HTLV-1 seronegative donors [1,8,21]. Despite initial data of inferior outcome in MUD allo-HSCT, more recent studies demonstrated the efficacy of MUD transplants in ATLL [8,28,29]. Since availability of unrelated donors is also limited, alternative donor sources like cord blood and haploidentical siblings have been used [8,12,17,22,27,30]. In the report of the Lymphoma Working Party of the European Bone Marrow Transplant Society (EBMT) on the pan-European experience during six years (2011–2016) on 17 patients, HSCT were performed from MRD (six cases), MUD (seven cases), haploidentical relative (three cases) and one unknown donor; no outcome data associated to donor source were reported [12]. In our cohort, two patients (25%) were transplanted from MRD and the other six (75%) from MUD. In one patient (no. 5) a second haploidentical transplant (haplo-HSCT) was performed after early relapse following the first MUD transplant. Four patients had survival longer than 2-years, three of them received transplant from MUD and one from MRD. The two patients that are still alive at the end of follow-up, at 41-months and 44-months, were transplanted from MUD and MRD, respectively. The haploidentical transplant using PT-Cy was performed as second transplant in a patient with uncontrolled disease with unfavorable outcome (death caused by disease progression). There are few data on the use of haploidentical donors in ATLL.

Yoshimitsu et al. published a retrospective analysis on outcome of haploidentical allo-HSCT without PT-Cy in patients with ATLL from the Japan Society for Hematopoietic Cell Transplantation database between 1985 and 2015. Forty-six patients were identified with an estimated 1- and 5-year overall survival rates of the entire cohort of 34.5% and 17.7%, respectively, but with high cumulative 1- and 5-year treatment-related mortality rates of 41.3% and 55.8%, respectively [30]. In the ATLL setting, more data on haploidentical transplants with PT-Cy are still needed. Considering the results obtained in other malignant hemopathies, we could expect that performing haplo-HSCT with PT-Cy earlier after diagnosis and in cases with controlled disease to achieve outcomes comparable to MUD transplantation procedures.

The role of a graft versus lymphoma (GVL) effect was highlighted by studies from Japan and Europe that have reported several aspects: superior OS after allo-HSCT compared to autologous transplants, and improved OS and reduced relapse rate in patients with limited acute and chronic GVHD, successful management of relapse by withdrawal of immunosuppression or by donor lymphocyte infusion (DLI) [12,21,24,26,27,28,31,32,33,34,35]. In our cohort, six patients (75%) developed GVHD: three (50%) acute GVHD and three (50%) chronic GVHD. Interestingly, out of the four patients that relapsed, all had GVHD (two presented acute GVHD and the other two presented chronic GvHD). Relapse occurred later in patients with chronic GVHD (7 and 25-months, respectively) compared to those with acute GVHD (2-months). The other two patients with GVHD (one acute and one chronic) died after complications associated to this condition. It is worth mentioning that the patient with chronic GVHD survived over three years without relapse. Moreover, both patients that are still alive after 41 and 44.2-months of follow-up did not develop any form of GVHD. This shows that the GVL effect is observed to a lesser extend in our cohort despite the results showing a similar incidence of GvHD in our patients compared to other studies [12,26].

In our cohort, the most frequent cause of death was represented by relapse; of the six patients who died during follow-up, four (66.6%) were disease-related deaths, whereas the other two (33.3%) deaths were related to treatment complications (one caused by metabolic imbalances and sepsis associated to chronic GvHD and one because of refractory acute GvHD). Compared to other studies, in our patient population the disease-related mortality was higher (66.6% vs. <48%) [8,12,22,26]. We analyzed if the high relapse rate could by associated to a lesser disease control determined by the conditioning type RIC or conditioning regimen used. In recent reports of Japanese and European groups RIC is used in most patients. The EBMT-LWP study reported the use of RIC in 13 patients (76%) and myeloablative conditioning (MAC) in three patients [12]. Ishida et al. published a retrospective study on 586 patients with allo-HSCT and demonstrated no difference in OS between MAC and RIC, with slightly higher TRM with MAC, but significantly higher disease-related mortality in RIC cases (*p* = 0.019) [22]. Although there are no prospective studies comparing different conditioning regimen, the same study published by Ishida et al. reported the superiority of fludarabine-based regimen combined with melphalan versus busulfan-based regimens in RIC setting [22]. The regimen we used, Flu/BU and TT/Flu/Bu are extensively used in lymphoid malignancies [36,37].

Another noticeable observation is the fact that three (60%) out of the five patients tested for HTLV-1 at 6-months post allo-HSCT presented a positive HTLV-1 viral load. This event occurred, although, at 30-days, all patients presented 100% donor chimerism and the donor samples were negative for HTLV-1. A possible pattern of viral load kinetic in transplanted patients is that it becomes initially undetectable but returns to detectable levels at 6 to 12-months post allo-HSCT [31]. Recurrence of HTLV-1 could predict relapse [32], but may also represent reinfection of donor lymphocytes, considering the high prevalence of HTLV-1 in Romanian population, with some patients maintaining remission and full donor chimerism despite reemergence of HTLV-1 [20,31,32]. In our study population, in the three patients with post-transplant HTLV-1 positivity, we registered a heterogenous evolution: one case had a post-transplant survival of more than 3-years without relapse and GHVD-related death; the second case had early relapse followed by a second haploidentical stem cell transplantation with unfavorable outcome (death caused by disease progression); in the third case relapsed occurred after 25-months leading to death through disease progression.

The limitations of the current study reside especially in the low number of included patients. This is due to the low incidence of the disease even in an endemic context and the low number of these patients that are eligible for HSCT. Another limitation of the study is represented by the context in which the HSCT was performed, as there are several treatment options that are not available in Romania. The strength of the current paper stands in the fact that it is the first paper to present the outcomes of ATLL HSCT patients in Romania, an endemic country for this disease.

## 4. Conclusions

The EBMT pan-European report published the results of allogeneic stem cell transplantation on 17 patients. Our case series is interesting as it presents the largest non-Asian cohort of ATLL transplanted patients outside the EBMT cohort. Overall, our results suggest that allo-HSCT improves survival in a subset of ATLL patients, particularly those with aggressive types. Allogeneic HSCT with RIC conditioning regimen represents a viable and potentially curative approach especially when performed in CR. Nonetheless, it must be considered that this therapeutic approach has its associated morbidities and mortality and further steps are needed to improve survival.

## Figures and Tables

**Table 1:** Clinical characteristics of patients.

Sex (Male/Female)	3/5
Median Age at Transplantation (Range)	39.5 (26–57)
Subtypes of ATLL Acute Lymphoma	1 7
Extranodal Involvement Skin Meningeal Multiple Organ Bone Marrow	7 3 1 1 5
Serum Calcium Levels (mg/dL) ≥11 <11	1 7
ECOG 0 1 2	3 3 2
Comorbidity Chronic Hepatitis Tuberculosis	3 1 2

ATLL—Adult T-cell leukemia/lymphoma; ECOG—Eastern Cooperative Oncology Group Performance Status.

**Table 2:** Patient treatment and outcome.

	Age	Sex	ATLL Type	Response to 1st Line Chemotherapy	Time Diagnosis HSCT (Month)	Disease Status at HSCT	Donor Type	Conditioning	GVHD Prophylaxis	GVHD	Relapse (Month after HSCT)	Status (Cause of Death)	Survival Post HSCT (Month)
1.	41	F	Lymphoma	No	23.5	CR	MUD	Flu/Bu	CsA + MTX	No	No	Alive	41
2.	40	F	Acute	No	15.5	SD	MUD	TT/Flu/Bu	CsA + MTX	Chronic	7	Dead (relapse)	12.3
3.	26	M	Lymphoma	Yes	11.5	CR (PET neg)	MSD	Flu/Bu	CsA + MTX	No	No	Alive	44.2
4.	57	F	Lymphoma	Yes	11	CR (PET neg)	MUD	Flu/Bu	CsA + MTX	Chronic	No	Dead (GVHD)	37.7
5.	37	F	Lymphoma	No	5.5	1: CR (PET neg) 2: PD	1: MUD 2: Haplo	1:TT/Flu/Bu 2: Flu/Cy/TBI	1: CsA + MTX 2:PT-Cy, Tacro, MMF	Acute, Grade 1 No	2 -	Dead (PD)	8.6
6.	33	M	Lymphoma	No	17.5	CR	MUD	Flu/Bu	CsA + MTX	Chronic	25	Dead (relapse)	26.7
7.	40	F	Lymphoma	Yes	5.5	CR (PET neg)	MUD	TT/Flu/Bu	CsA + MTX	Acute, Grade 4	No	Dead (GVHD)	2.3
8.	48	M	Lymphoma	No	5	SD	MSD	TT/Flu/Bu	CsA + MTX	Acute, Grade 1	2	Dead (relapse)	3.5

CR, complete remission; SD, stable disease; PD, progressive disease; MUD, matched unrelated donor; MSD, matched sibling donor; Haplo, haploidentical donor; Flu, fludarabine; Bu, Busulfan; TT, Thiotepa; Cy, Cyclophosphamide; TBI, total body irradiation; CsA, Cyclosporine A; MTX, Methotrexate; PT-Cy, post-transplant cyclophosphamide; Tacro, Tacrolimus; MMF, Mycophenolate mofetil; GVHD graft versus host disease; HSCT hematopoietic stem cell transplant; PET positron emission tomography.
